# Cas9-mediated excision of proximal DNaseI/H3K4me3 signatures confers robust silencing of microRNA and long non-coding RNA genes

**DOI:** 10.1371/journal.pone.0193066

**Published:** 2018-02-16

**Authors:** Harshavardhan Janga, Marina Aznaourova, Fabian Boldt, Katrin Damm, Arnold Grünweller, Leon N. Schulte

**Affiliations:** 1 Institute for Lung Research, Philipps University, Marburg, Germany; 2 Institute for Pharmaceutical Chemistry, Philipps University, Marburg, Germany; National Institutes of Health, UNITED STATES

## Abstract

CRISPR/Cas9-based approaches have greatly facilitated targeted genomic deletions. Contrary to coding genes however, which can be functionally knocked out by frame-shift mutagenesis, non-coding RNA (ncRNA) gene knockouts have remained challenging. Here we present a universal ncRNA knockout approach guided by epigenetic hallmarks, which enables robust gene silencing even in provisionally annotated gene loci. We build on previous work reporting the presence of overlapping histone H3 lysine 4 tri-methylation (H3K4me3) and DNaseI hypersensitivity sites around the transcriptional start sites of most genes. We demonstrate that excision of this gene-proximal signature leads to loss of microRNA and lincRNA transcription and reveals ncRNA phenotypes. Exemplarily we demonstrate silencing of the constitutively transcribed MALAT1 lincRNA gene as well as of the inducible miR-146a and miR-155 genes in human monocytes. Our results validate a role of miR-146a and miR-155 in negative feedback control of the activity of inflammation master-regulator NFκB and suggest that cell-cycle control is a unique feature of miR-155. We suggest that our epigenetically guided CRISPR approach may improve existing ncRNA knockout strategies and contribute to the development of high-confidence ncRNA phenotyping applications.

## Introduction

Since the decryption of the human genome sequence thousands of non-coding RNA (ncRNA) genes have been discovered. Meanwhile ncRNAs have been implicated in transcriptional and post-transcriptional control of major cellular processes, ranging from cell-cycle progression to lineage commitment and immune-responses. While microRNAs exhibit a size range of 20–22 nt and regulate gene expression through translational repression and destabilization of target transcripts [1], long non-coding RNAs (lncRNAs) are less uniform in function. By definition, lncRNAs are > 200 nt in size and lack coding sequences (CDS). LncRNAs have been suggested to interact with proteins to function e.g. as decoys, scaffolds or guides [2] and can be found both the nucleus and the cytosol.

For decades ncRNA loss-of-function studies in mammals were limited to small animal models. The discovery of Cas9 as an RNA-programmable DNA nuclease has enabled gene knockouts in human cells at low cost, opening a new chapter in genetics [3–5]. While functional knockouts of coding-genes are typically achieved though frame-shifting mutations within the CDS, little consensus exists regarding CRISPR/Cas9-strategies for silencing of non-coding RNA genes. Due to the lack of a CDS only in rare cases point-mutations within critical ncRNA regions can be expected to abrogate gene function.

Recently, functional ncRNA gene knockouts have been achieved by integration of RNA destabilizing elements [6] or excision of assumed promoter regions and exons by guiding Cas9 to two independent sites flanking the DNA sequence to be excised [7–10]. This strategy has generally proven itself successful, however, genomic deletions might affect neighbouring genes due to changes in local chromatin structure or redirection of the activity of distal enhancers. Furthermore, the provisional status of current ncRNA gene annotations may render it difficult to select essential promoter or exon regions to be knocked out. In the present work we introduce an approach, enabling ncRNA gene knockouts through excision of a defined transcriptional start site signature even in provisionally annotated loci, guided by epigenetic data.

Accessibility of eukaryotic genes for polymerases and transcription factors depends on the local chromatin condensation state, which is dynamically regulated through histone acetylation, methylation or phosphorylation marks. Histone H3-acetylation at lysine residues K9 and K27 (H3K9ac and H3K27ac) for instance is found in active gene regions, whereas tri-methylation of the same residues (H3K9me3 and H3K27me3) is a hallmark of transcriptionally repressed chromatin. However, histone methylation does not always lead to local chromatin silencing. H3K4me1 for instance marks active enhancers, while transcriptional start-sites (TSS) of active genes typically carry H3K4me3 tags. Gene-proximal H3K4me3 marks typically coincide with DNaseI hypersensitivity sites (HSS), indicative of accessible chromatin [11]. Overlaying DNaseI HSS and H3K4me3 ChIP-Seq data provides a means to identify the core TSS regions of human genes [11], even in provisionally annotated genomic regions.

Systematic mapping of chromatin modifications by the ENCODE project has established detailed histone modification maps in diverse human cell types. Here we made use of these publically available data to identify TSSs of microRNA and lncRNA genes for targeted excision by CRISPR/Cas9. We hypothesized that knockout of the TSS-associated DNaseI/H3K4me3 signature terminates ncRNA gene transcription. We evaluated our strategy exemplarily for one constitutively transcribed ncRNA gene (MALAT1), one obligate inducible ncRNA gene (miR146aHG) and one base-line expressed inducible ncRNA gene (miR155HG). MALAT1 is a highly expressed lncRNA, which has been implicated in diverse cellular processes such as alternative splicing, cell cycle progression and metastasis [12–14]. MiR-155 and miR-146a on the other hand have been described as regulators of inflammatory responses elicited on sensing of molecular pathogen signatures by pattern-recognition receptors (PRRs). A prototypic example for pattern recognition is the sensing of bacterial lipopolysaccharide (LPS) by the PRR Toll-like receptor 4 (TLR4), which triggers inflammatory gene expression through transcription factors such as NFκB, AP1 and IRF1-9. In mononuclear phagocytes, which are key players in mammalian antimicrobial immunity, miR-146 and miR-155 have been shown to confer negative-feedback control within PRR signalling [15–17]. However, miR-155 has also been suggested to promote inflammation under certain conditions [18, 19], therefore its role in the human innate immune response has remained controversial.

Here we show that excision of proximal DNaseI/H3K4me3 marks silences both inducible and constitutively transcribed ncRNA genes in human cells. Our results suggest a primary role of miR-146a and miR-155 but not MALAT1 in feed-back control or PRR-NFκB signalling in human monocytes and positive control of G2/M cell cycle phase accumulation as a unique function of miR-155. Our approach establishes a robust workflow for ncRNA gene silencing with minimal genome sequence alteration and may improve current CRISPR libraries for systematic loss-of-function screenings.

## Materials and methods

### Cell culture

U937 monocytes and Hek293 cells (ATCC) were grown in RPMI 1640 medium (Thermo Fisher), supplemented with 10% FBS (Biochrom) and 1% penicillin / streptomycin solution (Thermo Fisher). One day prior to experimental stimulations and readouts U937 cells were seeded into 12-well plates at 5×10^5^ cells/ml. Lipopolysaccharide (LPS) stimulations (LPS from *Salmonella* Typhimurium, Sigma Aldrich) for the indicated durations were carried out at a concentration of 1 μg / ml. For CRISPR-plasmid pre-validation Hek293 cells were seeded into 12-well plates at 4×10^5^ cells / ml one day prior to transfection.

### CRISPR/Cas9

**Fig 1:**
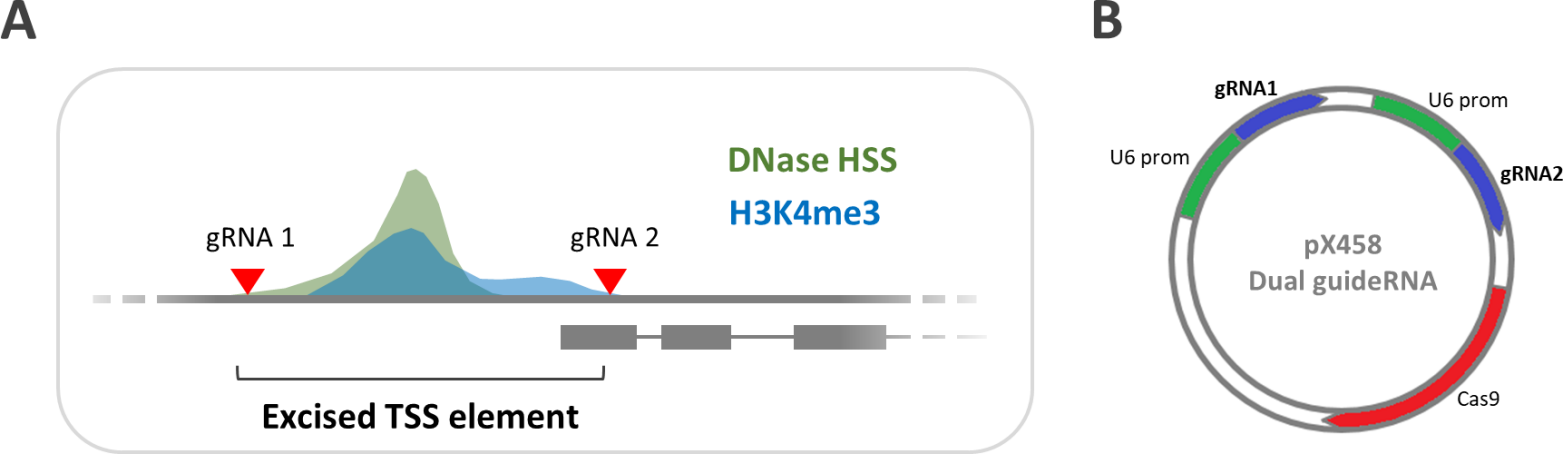
Silencing ncRNA genes based on proximal H3K4me3/DNaseI signatures. **A)** Schematic representation of the ncRNA knockout strategy. To abrogate transcription a signature consisting of H3K4me3 and DNaseI hyper-sensitivity (DNase HSS) signals overlapping at the gene proximal promoter is excised through two flanking guideRNAs. **B)** Cloning strategy to obtain a pX458 CRISPR vector construct simultaneously expressing two guideRNAs driven by repeated U6 promoters for knockout of a given ncRNA gene TSS signature. The depicted insert is generated by gene synthesis.

A synthetic DNA segment (Integrated DNA Technologies) introducing the first guideRNA and scaffold sequence, a second U6 promoter and the second guideRNA sequence (see Fig 1B and S7 Fig) was inserted into the BbsI site of the pX458 vector (Zhang lab [20], through Addgene). GuideRNAs flanking DNaseI/H3K4me3 sites were designed through crispr.mit.edu. For CRISPR construct pre-validation 1 μg of plasmid DNA per well was transfected into Hek293 cells using Lipofectamine 2000 (Thermo Fisher) according to the manufacturer’s protocol. For generation of homozygous U937 knockout clones cells were transfected with Lipofectamine 3000 (Thermo Fisher) according to the manufacturer’s instructions with 1 μg of plasmid DNA per/well, followed by centrifugation of culture plates for 2.5 h at 2000 rpm and 37°C to promote transfection. 24 hours later single transfected cells were sorted into the wells of a 96-well plate (transfected cells were detected through GFP, co-expressed from the pX458 vector) using an Aria III cell sorter (BD) with 100 μm nozzle. 96-well plates were pre-filled with RPMI 1640 complete medium, supplemented with 100 μg /ml Normocin (Invivogen) prior to sorting. During clonal expansion fresh medium was added every 5 days. Knockout events were detected by genomic DNA isolation (Nucleospin tissue kit; M&N) and genomic PCR with primers flanking the respective DNA-element to be deleted (S1 Table). Knock-out bands were verified by Sanger-sequencing (Seqlab GmbH, Göttingen). A step-by-step has been deposited at protocols.io: http://dx.doi.org/10.17504/protocols.io.mmic44e.

To analyse the distance of off-target sites to transcriptional start sites all genomic off-target sites predicted by crispr.mit.edu for each guideRNA were mapped to the GRCh38 reference genome annotation (ENSEMBL BLAST) and the distance from the 3’ end of the off-target site to next annotated transcript 5’ end was determined using ENSEMBL GRCh38 coordinates. To determine whether guideRNAs used in the present study are specifically prone to targeting of TSSs results were compared to random guideRNAs and their off-targets predicted by crispr.mit.edu. To this end, random genomic windows were obtained using R and the “sample” function with random determination of the chromosome number followed by the position on the selected chromosome and the strand. A 250 bp window starting from the obtained random genomic position was used as input for crispr.mit.edu and the top-ranking guideRNA and its predicted off-target positions were selected. Log2 distances of guideRNA off-target sites to annotated transcriptional start positions were calculated as follows: log2(1/distance [bp]) for off-target sites located upstream of an annotated transcriptional start site; log2(distance [bp]) for off-target sites located downstream of an annotated start site. Values were plotted against frequency for cumulative density plots (see S1 File).

### Real-time PCR

Real-time PCR was performed using the PowerUP^TM^SYBR® green master mix kit (applied biosystems) and a Quantstudio3 device (Applied Biosystems) according to the manufacturer’s instructions. RNA was extracted using the Trizol (Thermo Fisher) method. To remove contaminating DNA the nucleic acid pellet was incubated with DNaseI (Thermo Fisher) and RNase Inhibitor (Promega) for 30 min at 37°C, followed by extraction with PCI (Sigma Aldrich) and precipitation with 30:1 ethanol / 5M sodium acetate. Real-time PCR primer pairs are listed in S1 Table. Expression changes relative to U6 snRNA as an internal control were calculated using the 2^-DDCT method [21].

### Northern blot

60 μg of RNA per lane in Gel loading buffer II (Ambion) were denatured at 95°C for 5 min prior to loading. RNA was size separated on a 15% PAA, 8 M Urea TBE gel and transferred onto a positively charged nylon membrane (Roche) at 50 V for 50 min in TBE buffer at 4°C. After UV cross-linking (120 mJ / cm^2^) DIG-labelled DNA probes (S1 Table, labelled using the 2^nd^ Generation DIG-Tailing Kit [Roche]) were hybridized over night at 42°C in DIG EasyHyb Buffer (Roche). For development the DIG Wash and Block Buffer Set (Roche) and CDP-STAR (Roche) were used according to the manufacturer’s instructions. CDP-STAR signal was recorded using the Intas Chemostar Imager system.

### Flow cytometry

AnnexinV stainings were performed as described previosuly [22]. For cell cycle analysis cells were stained with propidium iodide (PI, Biolegend) with ethanol fixation. Briefly, cells were fixed with ice-cold 70% ethanol and incubated at– 20°C for 1 h. Cells were then washed with PBS and resuspended in staining buffer (PBS, 0.01% Tween, 20 μg / ml PI, 1 μl / ml RNaseA [100 mg / ml, Macherey Nagel]), incubated at 37°C for 30 min and washed once with PBS, followed by flow cytometry analysis. For NFκB p65 phosphorylation and ERK1/2 phosphorylation assays cells were fixed with 90% methanol, washed with PBS and resuspended in staining buffer (PBS, 0.1% FBS, 1 μl PE anti phospho-p65 [Ser529 phosphorylation; ThermoFisher # 12-9863-42] or 1 μl APC anti phospho-ERK1/2 [Tyr202/204; ThermoFisher # 17-9109-41]). On incubation for 1 hour at room temperature cells were washed with PBS, followed by data acquisition. Changes in p65-signal were determined through the yellow channel geo-mean. Data were recorded using a Guava easyCyte flow cytometer (Millipore). FCS3.0 files were analysed using Flowing Software (http://flowingsoftware.btk.fi/).

### RNA-Seq & ChIP-Seq analysis

Demultiplexed RNA-Seq reads were downloaded from the NCBI GEO database (dataset SRA numbers see S2 Table). SRA archive files were converted into fastq file-format using the SRA-toolkit. Reads were mapped to the GRCh38 human reference annotation using the CLC Genomics Workbench (Qiagen) with standard settings (mismatch cost = 2; insertion cost = 3; deletion cost = 3; length fraction = 0.8; similarity fraction = 0.8). For co-expression analysis datasets (see S2 Table) were filtered for genes with RPKM ≥ 1 under at least one condition. For ncRNA expression analysis (S1 Fig) monocyte datasets (see S2 Table) were filtered for genes with an average RPKM across all libraries ≥ 1. ENCODE primary human monocyte H3K4me3, H3K27me3 and DNaseI HSS datasets were downloaded from the NCBI GEO database. Accession numbers: DNaseI HSS data: SRR608865, SRR608866; H3K4me3 data: SRR568364, SRR568365; H3K4me27 data: SRR568417, SRR568418. Reads were mapped to the GRCh38 human reference genome sequence using the CLC Genomics Workbench with standard settings. BAM-files were sorted and indexed using SAMtools and coverages were visualized using the Integrative Genomics Viewer [23].

### Co-expression & KEGG pathway analysis

To identify genes co-expressed with miR-146a or miR-155 host-genes or MALAT1 the RPKM values for the respective gene in all analysed NCBI GEO datasets (S2 Table) were linearly projected and RPKMs of all coding genes in the datasets were projected on the same axis. R^2^ was calculated (Excel) to determine co-expressed genes. Using ENSEBL gene IDs from all genes with R^2^ values ≥ 0.65 (miR-146a, miR-155 or MALAT1 co-expressed genes) KEGG pathway analysis and expression-network analysis was done using ConsensusPathDB [24] and the gene set “over-representation” and “induced network modules” tools. KEGG pathway analysis was limited to the top 10 pathways, except for MALAT1 (only one pathway hit). Induced network module analysis outputs are shown in S5 Fig.

### Step-by-step protocol

A step-by-step protocol describing the mammalian non-coding RNA gene knockout procedure established by the present report has been deposited on protocols.io and can be accessed through: http://dx.doi.org/10.17504/protocols.io.mmic44e

## Results

### Knockout strategy based on epigenetic signatures

An epigenetic hallmark of active gene proximal promoters is H3K4 tri-methylation, which often co-insides with open (DNaseI-sensitive) chromatin (Fig 1A). Aiming at a robust ncRNA gene knock-out strategy with minimal genomic sequence ablation we determined whether excision of this gene-proximal signature is sufficient to terminate transcription. To this end, for each gene locus to be knocked out the existing pX458 CRIPSR vector [20] was equipped with expression cassettes for two guideRNAs, cleaving before and after the proximal DNaseI/H3K4me3 site of the selected ncRNA gene (Fig 1A and 1B).

**Fig 2:**
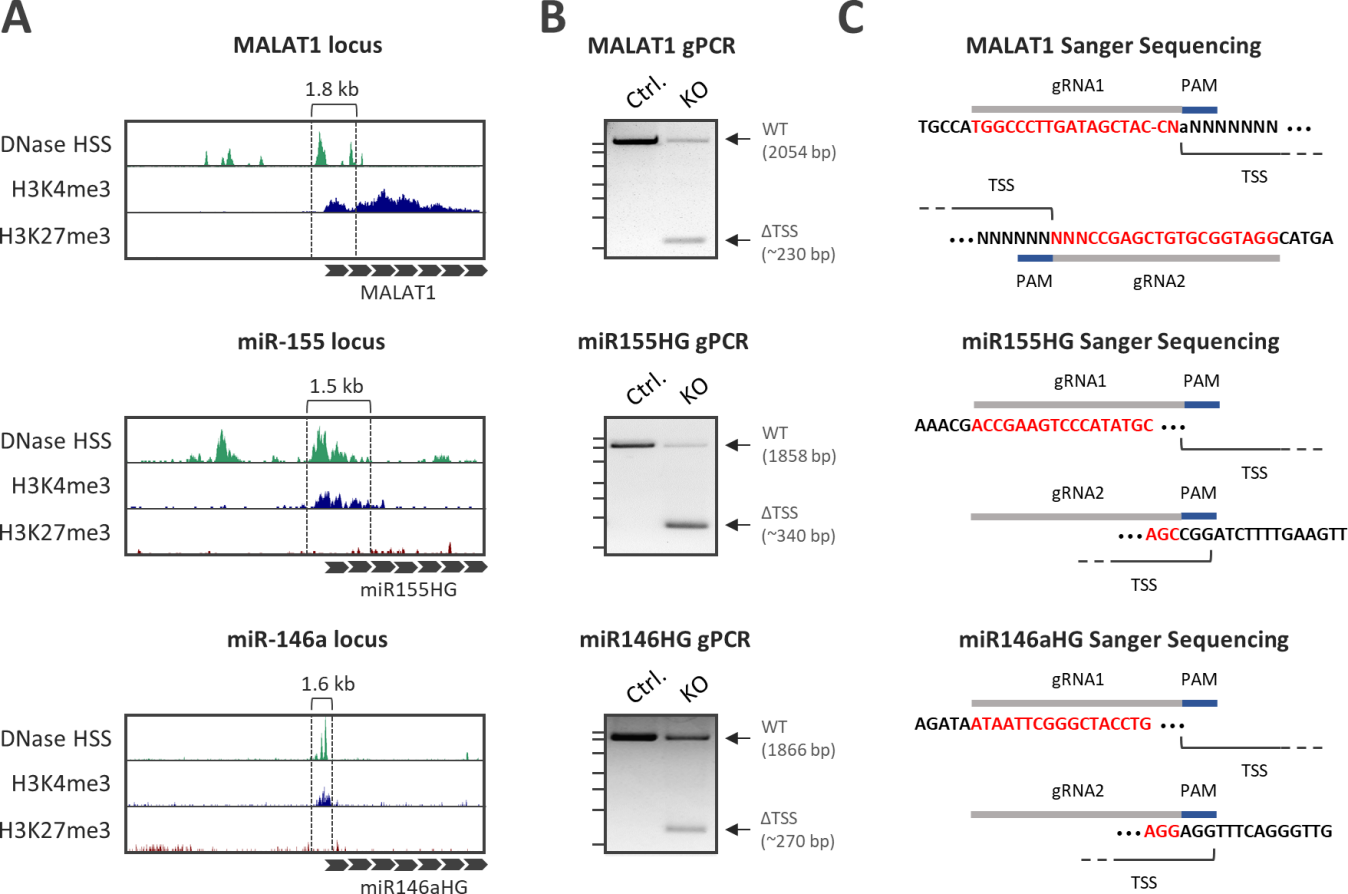
Excision of H3K4me3/DNaseI signatures by dual guideRNA CRISPR vectors. **A)** Primary human monocyte DNase- and ChIP-Seq (H3K4me3 and K3K27me3) coverages at the transcriptional start sites of the MALAT1, miR-146a and miR-155 ncRNA genes. Regions selected for targeted excision are marked (dashed lines). **B)** Genomic PCR with primers flanking the respective H3K4me3/DNaseI element to be excised. Besides the wild-type band (WT) a band of reduced size (ΔTSS) is detected after transfection of HEK293 cells with the respective ncRNA knockout CRISPR construct (KO) but not after transfection of a control CRISPR construct (Ctrl.). Position of the DNA ladder bands (2.5 kb, 2 kb, 1.5 kb, 1 kb, 750 bp, 500 bp, 250 bp) is indicated to the left of each gel image. **C)** Sanger-sequencing of ΔTSS bands validates the deletion in the MALAT1, miR155 and miR146a promoter, respectively. Positions of guideRNAs (gRNA) and protospacer-adjacent motifs (PAM) are indicated.

To test our strategy experimentally we chose two well-established innate immune genes highly induced by microbial signatures in human monocytes (miR146aHG, miR155HG; S1 Fig). Furthermore, the MALAT1 lncRNA gene was chosen to test whether our approach may blunt the expression of ncRNAs constitutively transcribed at prominent levels (S1 Fig). Analysis of primary human monocyte ChIP-Seq data revealed H3K4me3 signatures overlapping with DNaseI hyper-sensitive sites around the annotated transcriptional start sites of all three genes (Fig 2A). Repressive H3K27me3 signals as a control were not enriched in the same gene regions (Fig 2A). To address the specificity of our approach we analyzed whether the guideRNAs designed to target the TSS signatures of miR146aHG, miR155HG or MALAT1, respectively, are prone to off-target binding of transcriptional start regions of unrelated genes. To this end, the distance of each predicted off-target site to the next annotated transcript 5’ end was determined, and results were visualized as cumulative density plots. No general enrichment of off-targets near annotated transcriptional start regions compared to a set of control guideRNAs targeting randomly selected genomic sequences was observed (S2A and S2B Fig). Rather, off-target sites were underrepresented in a region of ~1000 bp up- and down-stream of annotated start sites (S2B Fig). Thus, our approach is unlikely to be biased towards off-target mutation of unrelated transcriptional start sites.

Prior to the generation of homozygous knockout clones, excision of the desired DNA segments by the CRISPR constructs was pre-validated by transfection into Hek293 cells and genomic PCR. Primers flanking the respective DNaseI/H3K4me3 sites amplified the expected wild-type bands from control-transfected cells. Additionally, shortened PCR products were obtained from cells transfected with the ncRNA knockout constructs, indicative of successful excision of the H3K4me3 signatures (Fig 2B). Sanger sequencing confirmed DNA-cleavage close to the predicted PAMs of the respective guideRNAs (Fig 2C). Thus, proximal DNaseI/H3K4me3 signatures of ncRNA genes can be readily identified using public epigenetic datasets and knocked out using a modified CRISPR vector expressing two guideRNAs simultaneously.

### Excision of H3K4me3 signatures abolishes ncRNA gene expression

**Fig 3:**
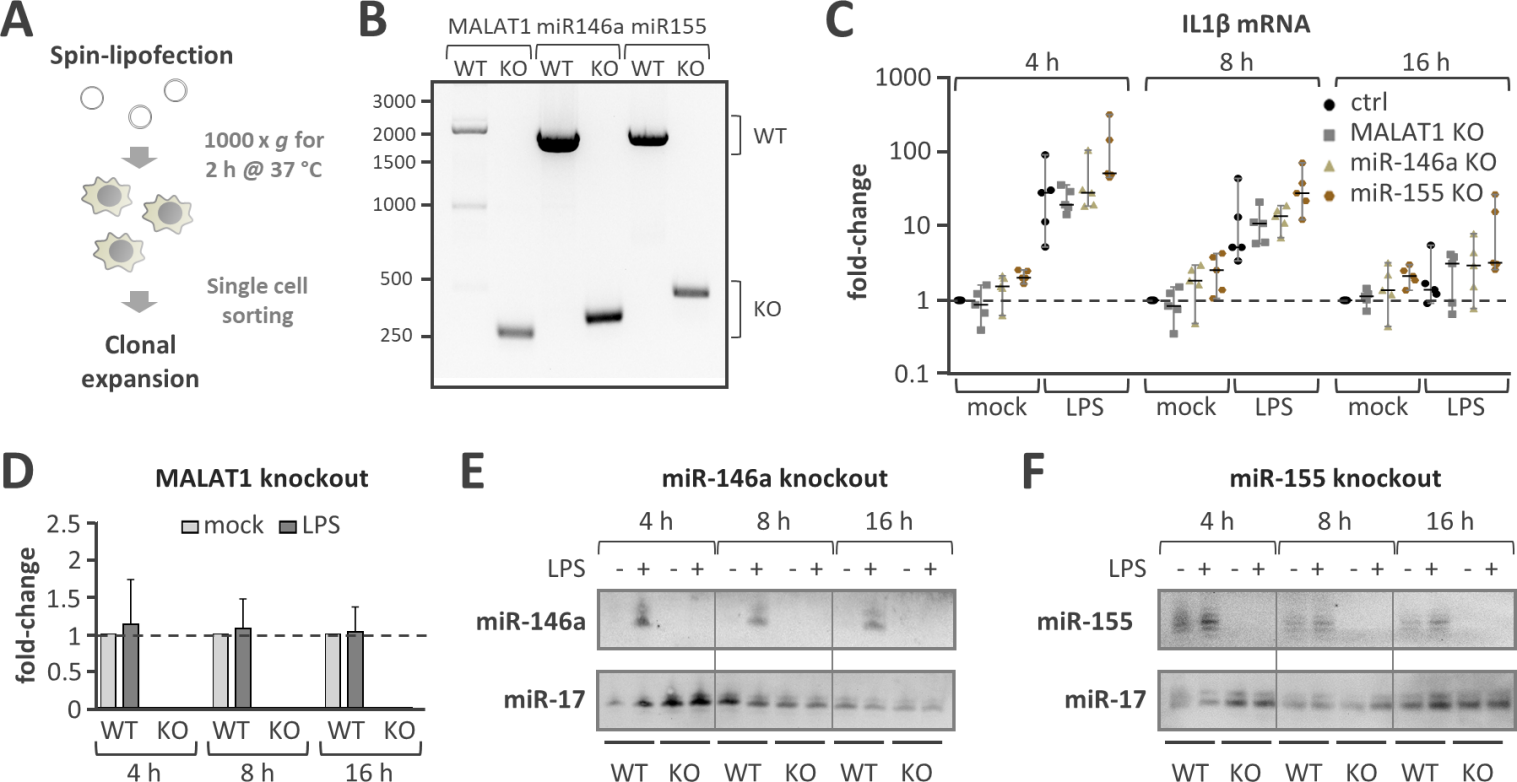
Excision of H3K4me3/DNaseI signatures abolishes ncRNA expression. **A)** Experimental overview. U937 monocytes are spin-lipofected with dual guideRNA CRISPR constructs and clonally expanded after single cell sorting of cells positive for GFP (co-expressed from the CRISPR vector). **B)** Genomic PCR showing homozygous deletion of the proximal promoter elements identified in Fig 2 of the MALAT1, miR155, miR146a genes after clonal expansion of monocytes transfected with the respective dual guideRNA CRISPR constructs (KO). Transfection of control CRISPR construct does not result in the deletion, respectively (WT). **C)** Real-time PCR analysis of IL1β mRNA expression in wild type (WT) and MALAT1, miR-146a or miR-155 TSS knockout monocytes after control-stimulation (mock) or activation with 1 μg / ml of LPS for 4h, 8h or 16h. Fold-changes compared to mock-stimulation of control cells are shown. **D)** Real-time PCR analysis of MALAT-1 expression in wild-type (WT) or in MALAT1 H3K4me3/DNaseI element deficient cell clones stimulated as in C). **E)** Northern blot with miR-146a, and miR-17 detection probes showing loss of miR-146a induction on stimulation with LPS (1 μg / ml) in monocytes deficient in the gene-proximal H3K4me3/DNaseI element (KO) but not in wild-type (WT) cell clones. **F)** Same as E) but with miR-155 gene proximal H3K4me3/DNaseI element excised and miR-155 instead of miR-146a detection.

To test whether deletion of proximal DNaseI/H3K4me3 signatures terminates ncRNA gene transcription CRISPR constructs from Fig 2 were transfected into human monocytic cell line U937. Monocytes are difficult to transfect with plasmid DNA, complicating genetic manipulation of these cells. We developed a spin-lipofection protocol, combining transfection with centrifugation to promote uptake of complexed CRISPR vector DNA. This was followed by single cell sorting and expansion (Fig 3A). Homozygous knockout cell clones were identified by genomic PCR (Fig 3B). For the MALAT1 gene knockout out of 36 tested clones 5 were identified as heterozygous and 3 as homozygous with regard to the TSS deletion. To obtain miR-146a knockout cells 37 clones were screened and 6 were found heterozygous while another 6 carried the deletion on all alleles. For miR-155 knockout cells out of 4 clones tested 1 was deficient in the TSS element on all alleles.

Induction of the mRNAs of IL1β (Fig 3C), IL8 and CCL4 (S3 Fig) as positive controls for the activation of monocytes by TLR4 agonist LPS was observed in all analysed knockout cell clones, ruling out major side-effects of the Cas9 system on cellular immune-responsiveness. Induction of all three immune-response markers was slightly elevated at 4 and 8 hours post stimulation in LPS-stimulated miR-155 deficient compared to control cells, this difference however did not turn out significant at the P < 0.05 level (one- and two-way ANOVA). Importantly, real-time PCR confirmed robust silencing of the highly transcribed non-coding RNA MALAT1 in control-treated or LPS-activated monocytes lacking the gene-proximal DNaseI/H3K4me3 signature (Fig 3D). Northern-blot analysis detected the expression of miR-146a exclusively upon LPS stimulation and an LPS-mediated increase in the expression of miR-155 in wild-type monocytes (Fig 3E and 3F). On the other hand, expression of these miRNAs was lost in cells deficient in the respective gene proximal DNaseI/H3K4me3 element (Fig 3E and 3F). Taken together, these observations demonstrate that excision of proximal DNaseI/H3K4me3 signatures is sufficient to silence ncRNA gene expression. Thus, our strategy provides a robust knock-out approach with minimal genomic sequence alteration based on a ubiquitous gene-proximal element.

### Identification of ncRNA knockout phenotypes

H3K4me3 signatures are readily detectable in front of the transcriptional start sites of most active human genes, including ncRNA genes [11, 25]. Therefore, systematic deletion of these signatures might enable robust ncRNA phenotypic screening. To provide proof-of-concept for the identification of ncRNA phenotypes using our knockout procedure we carried out computational predictions followed by wet-lab experimental validations using the ncRNA knockout cells generated above.

To narrow down experimental assays for phenotype identification we predicted miR-146a, miR-155 and MALAT1 functions by co-expression network analysis. To this end, 45 published datasets from control- and immune-stimulated mononuclear phagocytes (S2 Table) were mined for co-expressed mRNAs, which were subjected to KEGG pathway and transcriptional network analysis. In line with the known roles of miR-146a and miR-155 our predictions implicated both microRNAs in inflammatory gene expression downstream of PRRs (S4A and S4B Fig). MiR-146 has been extensively characterized as a TLR negative feedback regulator. On the other hand, it had remained debated, whether miR-155 primarily promotes or limits inflammatory responses downstream of PRR activation [19, 26]. Our computational analysis furthermore predicted miR-155 but not miR-146a to be associated with cell-cycle regulation (S4B Fig). MALAT1 co-regulated genes on the other hand displayed only weak functional associations, and “Transcriptional misregulation in cancer” was the only identified KEGG pathway term (S4C Fig). To address our predictions experimentally we monitored phosphorylation of NFκB and ERK1/2, which occurs downstream of TLR4 activation, and carried out cell cycle and apoptosis assays with control-treated and LPS-activated wild-type and knockout monocytes.

**Fig 4:**
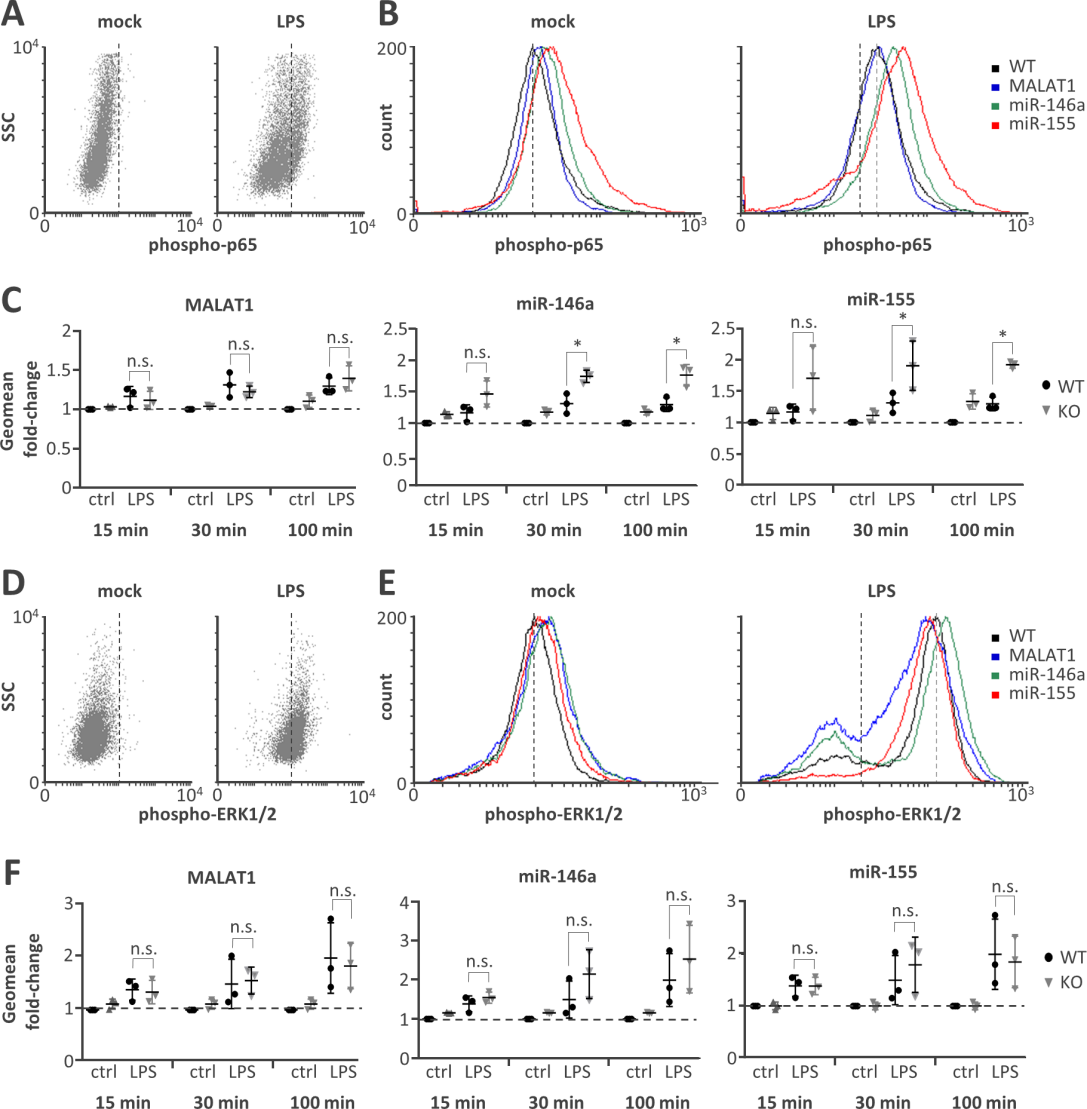
Elevated NFκB p65 but not ERK1/2 activity on miR-146a and miR-155 knockout. **A)** Representative FACS scatter plots showing a right-shift of 30 min LPS-stimulated (1 μg / ml) compared to mock-treated monocytes stained with phospho-p65 antibody (PE-channel). **B)** Representative histogram plots showing an increased right-shift of miR-146a and miR-155 deficient compared to control or MALAT1 deficient monocytes after 30 min LPS-stimulation (1 μg / ml) and staining with a phospho-p65 antibody (PE-channel). **C)** Fold change in phospho-p65 signal in monocytes stimulated with LPS (1 μg / ml) for 15, 30 or 100 min compared to mock-treatment (ctrl) in wild-type (WT) or the indicated ncRNA knockout (KO) cells. All fold-changes are relative to the respective WT mock control. **D-F)** Same as A-C) but with phospho-ERK1/2 staining (APC-channel). Statistical significance was determined by a one-way ANOVA test with multiple comparisons (* p<0.05).

**Fig 5:**
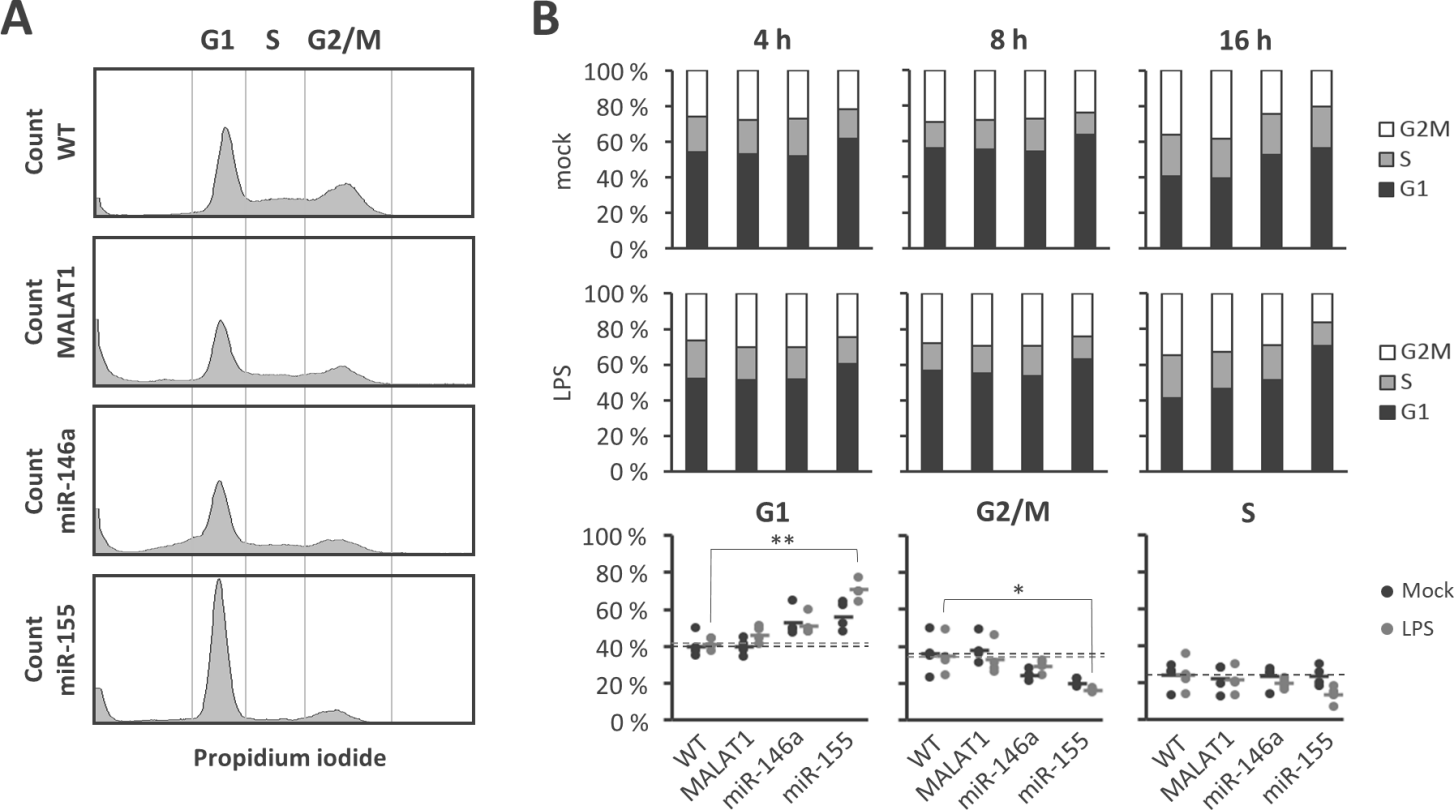
Knockout of miR-155 affects monocyte cell cycle. **A)** Gating scheme for determining cell fractions in G1/G0-, S- or G2/M-phase through propidium iodide staining. Representative data from LPS-treated wild-type (WT) or ncRNA knockout monocytes are shown. **B)** Quantification of percent-distribution of cell fractions in G1/G0-, S- or G2/M-phase in 4, 8 or 16 h mock or LPS (1 μg / ml) treated monocytes (upper and middle panels) and representation of individual mean- and replicate values for the 16 h time-point (lower panel). Data refer to wild-type (WT), MALAT1, miR-146a and miR-155 knockout U937 cells as indicated below the lower panel. Statistical significance was determined by one-way ANOVA with multiple comparisons (* p<0.05, ** p<0.01).

**Fig 6:**
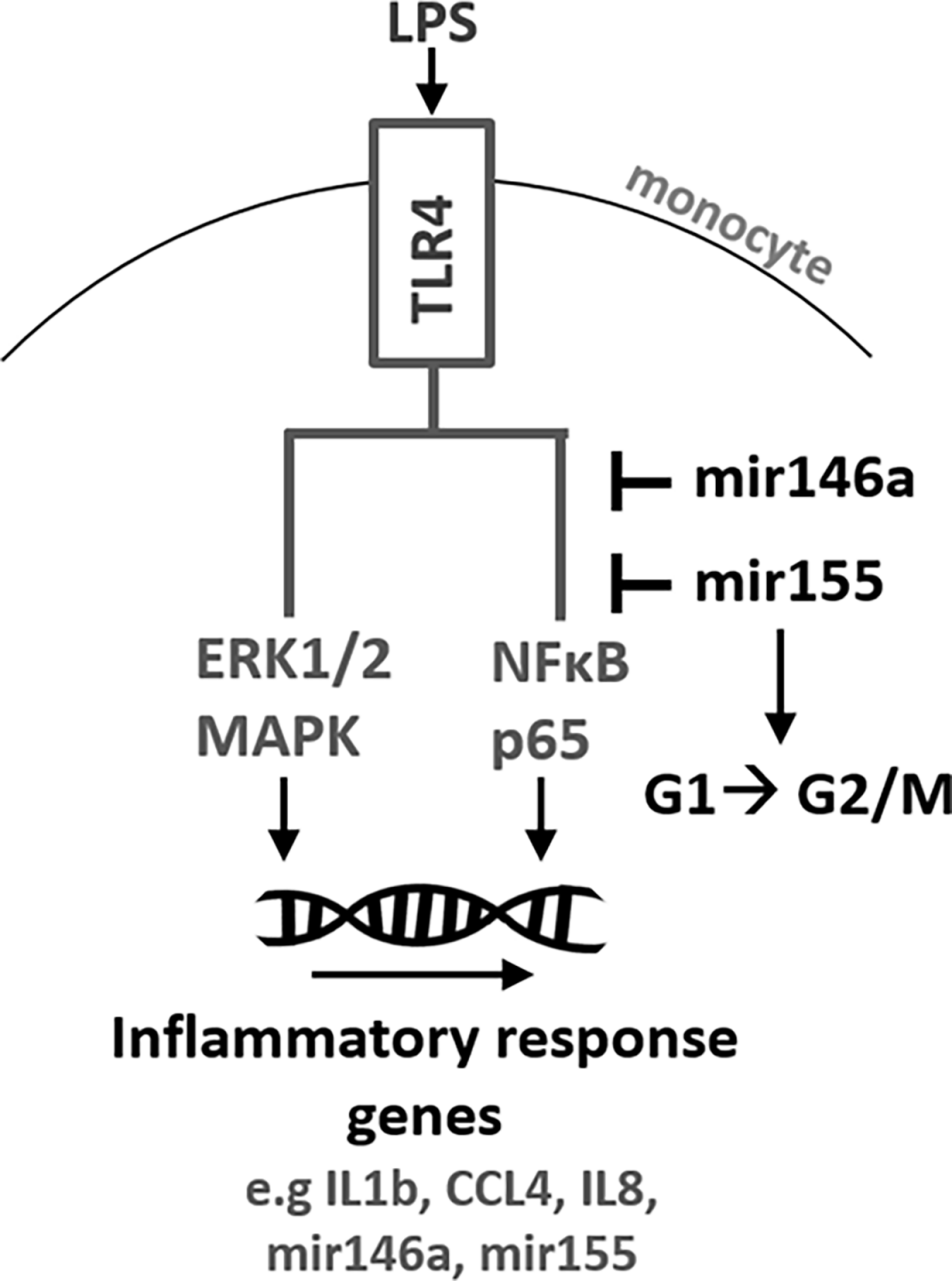
Model summarizing the observed ncRNA knockout phenotypes. NFκB p65 phosphorylation data indicate a negative feed-back function of both miR-146a and miR-155 in TLR signaling, while ERK1/2 signaling is not affected. Promoting entry into the G2/M cell cycle phase is a function unique to miR-155.

As a measure for the transcriptional activity of NFκB we determined changes in Ser592 phosphorylation of the p65 subunit by intracellular staining and flow-cytometry (Fig 4A). Importantly, NFκB p65 phosphorylation was significantly elevated in both miR-146a and miR-155 deficient cells compared to wild-type cells at 30 and 100 min post onset of the LPS-stimulus (Fig 4B and 4C, middle and right panel). In MALAT1 deficient cells however NFκB p65 phosphorylation was indistinguishable from wild-type cells (Fig 4B and 4C, left panel). This suggests that both miR-146a and miR-155 but not MALAT1 act as negative feedback regulators of TLR-NFκB signalling and confirms our previous results with murine cells [15]. In a similar FACS-based assay (Fig 4D and 4E) no impact of MALAT1, miR-146a or miR-155 ablation on LPS-stimulated ERK1/2 phosphorylation was observed (Fig 4E and 4F). Thus, the increased phosphorylation of NFκB in miR-146a and miR-155 deficient cells does not represent a generalized effect on TLR4-mediated signalling cascades. Furthermore, confirming our predictions, knockout of miR-155 but not miR-146a lead to a substantial shift of monocytes out of G2/M and into G1/G0 cell cycle phase compared to wild-type cells (Fig 5A and 5B), in line with the known implications of miR-155 in cancer [27]. No effect of MALAT1 knockout on the cell-cycle was observed (Fig 5A and 5B), despite its classification as a cancer-related gene [28]. Furthermore, apoptosis rates as determined by AnnexinV-staining were similar in all cell clones (S6 Fig), suggesting that none of the examined ncRNAs is specifically involved in cell death and ruling out broad side-effects of our CRIPSR approach on cell viability. In summary our ncRNA knockout method validates both miR-146a and miR-155 as negative feed-back regulators of the pro-inflammatory TLR-NFκB axis in human monocytes. Our results furthermore suggest that cell-cycle control is a unique feature of miR-155, functionally discriminating this miRNA from co-regulated miR-146a (Fig 6).

Taken together, our study establishes a robust lincRNA and microRNA knockout method and demonstrates that excision of TSS-associated DNaseI/H3K4me3 chromatin marks is sufficient to silence both constitutively expressed and inducible genes. We provide a detailed step-by-step protocol enabling ncRNA gene knockouts even in cells regarded difficult to manipulate (http://dx.doi.org/10.17504/protocols.io.mmic44e). Our robust workflow may facilitate future phenotypic screening approaches to characterize the many yet poorly studied ncRNA loci in mammalian genomes.

## Discussion

Our study introduces an improved method for the generation of ncRNA gene knockouts. While previous strategies have relied on individual determination of essential gene regions we demonstrate that excision of DNaseI/H3K4me3 signatures, which constitute universal TSS hallmarks, is sufficient to confer robust gene silencing.

As a general note of precaution, excision of genomic DNA segments may affect expression of neighbouring genes. In the case of bidirectional transcription for instance, deletion of promoter segments may silence transcription from both DNA strands, rendering it difficult to assign an observed phenotype to one specific transcript. We believe, that our approach minimizes those non-specific effects by excising a strand-specific TSS element rather that the entire promoter. Nevertheless, unwanted side-effects on transcription of neighbouring elements must be ruled out experimentally in any case where multiple transcripts originate from the same genomic locus. Furthermore, off-target effects of the guideRNAs used to ablate the TSS of a gene must be considered as well as targeting of unrelated TSSs. Our computational analysis suggests that TSS regions are underrepresented among guideRNA off-target sequences, in line with the relatively small fraction of the human genome sequence being occupied by TSS [29]. Nevertheless, careful evaluation of the selected guideRNAs (see step-by-step protocol) and exclusion of guideRNA pairs with shared off-target sites in proximity to a gene sequence is recommended. In addition, high ranking predicted off-targets may be amplified by genomic PCR and analysed for mutations by Sanger sequencing, as exemplarily carried out for the ncRNA knockout constructs used in the present study (S2C Fig). In our case no off-target mutations in the inspected off-target loci were observed.

While this manuscript was in preparation a study by Pulido-Quetglas et al. suggested that deletion of lncRNA enhancer elements may reduce downstream gene expression [30]. This strategy however may be less universally applicable since enhancers may be located far away from the gene proximal promoter and thus hard to assign exclusively to a given gene. Furthermore, enhancers may give rise to functional lncRNAs themselves [31] and therefore their deletion might provoke additional effects uncoupled from the silencing of immediate downstream genes. We predict that side effects, inherent to every CRISPR-based gene knockout method, are less likely to occur with our targeted TSS excision approach than with large promoter or enhancer deletions. Furthermore, Engreitz et al. recently showed that lincRNA promoter deletions reduce lncRNA expression more effectively than individual intron, exon or splice-site deletions [32]. Still, due to the frequent bidirectional and overlapping transcription in mammalian genomes, many genes might not be suited for CRISPR-based loss-of-function experiments [33]. In those cases, antisense-inhibition approaches using RNAi or Gapmer technology may stay the primary methods of choice and in any case phenotypes obtained through CRISPR/Cas9 based approaches should be validated using independent methods. We suggest, that our improved ncRNA knockout strategy may be primarily used in intergenic ncRNA loss-of-function experiments, where a given TSS can be unambiguously assigned to one individual gene. Recently, thousands of intergenic long non-coding RNA genes (lincRNAs, including many microRNA host-genes) have been identified in the human genome [34] and most of those genes are still of unknown function. We believe that our method can make a significant contribution to the functional characterization of this major ncRNA contingent of the human transcriptome.

Exemplarily we have knocked out the constitutively expressed MALAT1 gene and the TLR-inducible genes miR146HG and miR155HG. Our observations suggest a primary function of both miR-146a and miR-155 in negative feedback control of NFκB signalling in human cells. This confirms previous observations reported by us and others [15–17, 35]. However, miR-155 has also been suggested to promote inflammatory responses through inhibition of SOCS1 and SHIP1 [18, 19]. While we cannot rule out that miR-155 may exert both feed-back and feed-forward control of TLR-signalling in a time- and stimulus-dependent manner or even simultaneously our observations suggest that the negative feedback function dominates in human monocytes. Interestingly, while the effect of both miRNA deletions on NFκB p65 phosphorylation was highly significant, the same trend was observed for IL1b, IL8 and CCL4 mRNA induction, however did not reach significance levels. This may indicate compensatory mechanisms acting on NFκB target genes when miR-146a and miR-155 feed-back regulation is absent, preventing from excessive immune gene activation. Furthermore, our observation that ERK1/2 phosphorylation is not affected by miR-146a or miR-155 ablation might indicate that these microRNAs primarily impact on the expression of genes which strictly depend on NFκB and are not co-regulated by the ERK-MAPK pathway. Finally, since we silenced the microRNA host genes the possibility of lincRNA functions independent of the miRISC pathway contributing to the observed phenotypes cannot be ruled out and should be addressed by future studies. The observation that MALAT1 knockout does not impact NFκB p65 phosphorylation or cell cycle progression confirms that our observations are not due to a general side-effect of the Cas9 enzyme on cellular immune responsiveness. On the other hand, the role of the highly abundant lncRNA MALAT1 in mononuclear phagocytes remains elusive, in line with other recent observations, reporting the absence of obvious phenotypes in MALAT1 deficient mice [36–38].

In summary, our ncRNA gene knockout approach using epigenetic information to narrow down essential TSS elements confers robust gene silencing. We provide proof-of-concept for the identification of ncRNA knockout phenotypes using well-established ncRNA loci. Besides improved generation of stable gene knockouts we suggest that our approach may complement existing CRISPR libraries for high-confidence intergenic non-coding RNA silencing in phenotypic screening assays.

## Supporting information

S1 Fig RNA-Seq analysis of long intergenic non-coding RNA expression in human monocytes. Plotted are average RPKMs across all conditions (Y-axis) and fold-changes (X-axis) of long intergenic non-coding RNA genes (including microRNA host-genes) derived from RNA-Seq analysis of monocytes either control-treated or stimulated with LPS, Listeria monocytogenes or LPS + Interferon-γ (NCBI GEO data, see S2 Table). Fold-changes from all three stimulations were averaged. MALAT1, miR146aHG and miR155HG data-points are highlighted.
(PDF)

S2 Fig GuideRNA off-target analysis. **A)** Upper panel: total number of off-targets (“off-targets”), and number of genic off-targets (“genic”) predicted by crispr.mit.edu for the guideRNA pairs (guide # 1 and 2) used to knockout miR146aHG, miR155HG or MALAT1. Lower panel: same as upper panel but for randomly selected guideRNAs number 1–6. **B)** Cumulative density plot representing the distance of predicted off-targets from A) to the next annotated transcript 5’ end (“TSS center)”. The guideRNA pairs for knockout of miR146aHG, miR155HG or MALAT1 are color-coded as indicated to the right (dashed lines). Random control guideRNA analysis are presented as grey dashed lines. The result of the combined analysis with data from all six guideRNAs targeting the miR146aHG, miR155HG or MALAT1 loci is shown as a solid black line (“Consensus ncRNAs”) and the combined analysis for all six random guideRNAs (“Consensus random”) as a solid grey line. **C)** Sanger sequencing results for the top predicted off-target loci of each guideRNA of the MALAT1, miR146aHG and miR155HG knockout constructs. Alignments aggregate the guideRNA sequence and the sequencing results obtained using wild-type or the respective knockout cells. The PAM motif is highlighted. Mutations are expected to occur ~3–4 nucleotides away from the PAM.
(PDF)

S3 Fig Real-time PCR analysis of IL8 and CCL4 expression in wild-type and ncRNA knockout cell clones. **A)** Analysis of IL8 mRNA expression changes in wild-type (“ctrl”) and the indicated ncRNA knockout cell clones comparing mock treatment and LPS stimulation (1 μg / ml) for the indicated durations. **B)** Same as A) but measuring CCL4 instead of IL8 mRNA expression changes.
(PDF)

S4 Fig In silico prediction of ncRNA functions in mononuclear phagocytes. **A)** Transcription factor network (left panel; transcription factors: bold; transcription factor target genes: grey) and KEGG pathway analysis (right panel) of mRNAs co-expressed with the miR-146a host transcript in 45 mononuclear phagocyte RNA-Seq experiments (see S2 Table). Each square in the pathway heat-map represents a gene included in the respective pathway. Pathways are shown in different colours. Only pathways covering ≥ 5 genes from the input list were visualized. **B)** Same as A) but with miR-155 host transcript co-expressed mRNAs. **C)** Same as A) but with MALAT1 co-expressed mRNAs. Only one pathway (covering 2 genes from the input list) was identified.
(PDF)

S5 Fig ConsensusPathDB output of the co-expression network analysis summaries shown in S1 Fig. Outputs of the “induced network” analysis for miR-146 host transcript (A), miR-155 host transcript (B) and MALAT1 host transcript (C) co-expressed mRNAs are shown. ConsensusPathDB legend is shown below the three co-expression network panels.
(PDF)

S6 Fig AnnexinV staining of control and knockout cell clones. The percentage of AnnexinV (AnxV) positive cells either control-treated or stimulated with LPS (1 μg / ml) for 16 hours is shown. Genetic backgrounds are indicated above the panel (WT = wild-type).
(PDF)

S7 Fig Dual guideRNA cloning cassette. The sequence of the dual guideRNA pX458 cloning cassette used in the present study is provided. The functional elements of the cassette, to be cloned into the pX458 CRISPR vector via BbsI sites, are color-coded according to the explanations given below the sequence. GuideRNA sequences to be inserted are shown as “NNN…” and highlighted in yellow.
(PDF)

S1 Table DNA oligos used in the present study. DNA oligos used for qPCR, genomic PCR, Northern blot detection and Sanger sequencing are listed.
(PDF)

S2 Table External RNA-Seq data with NCBI GEO database accession numbers. All RNA-Seq datasets deposited in the NCBI GEO pipeline and used in the present study are listed.
(PDF)

S1 File Analysis of guideRNA off-target distance to annotated transcripts. Spreadsheet containing the data to calculate the distances of guideRNA off-targets to annotated transcript 5’ ends.
(XLSX)
