# Supplementary figures and images for: Cas9-mediated excision of proximal DNaseI/H3K4me3 signatures confers robust silencing of microRNA and long non-coding RNA genes
Source: PLoS One. 2018 Feb 16;13(2):e0193066. doi: 10.1371/journal.pone.0193066 (PMC5815609; doi:10.1371/journal.pone.0193066)

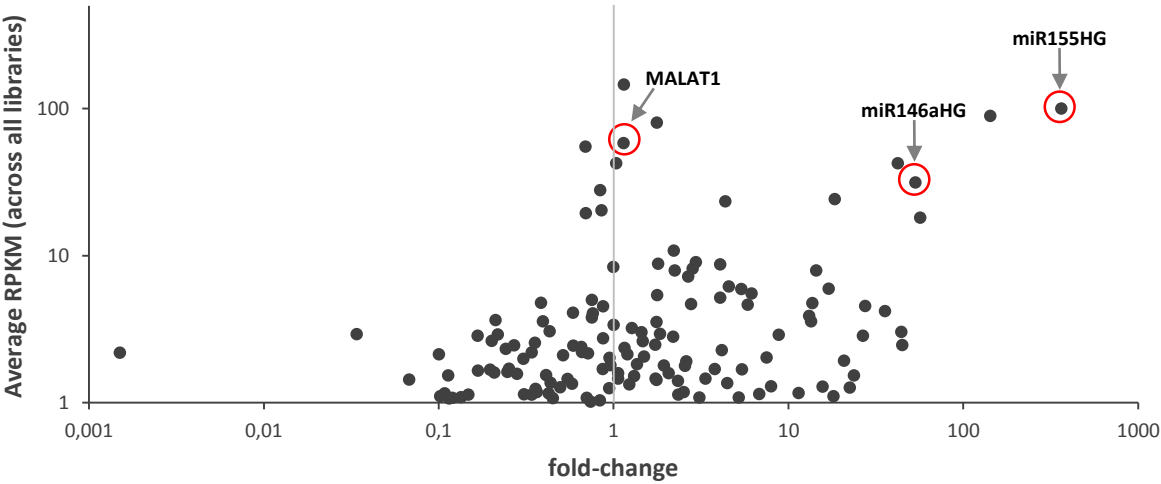

Supplement: S1 Fig — Plotted are average RPKMs across all conditions (Y-axis) and fold-changes (X-axis) of long intergenic non-coding RNA genes (including microRNA host-genes) derived from RNA-Seq analysis of monocytes either control-treated or stimulated with LPS, Listeria monocytogenes or LPS + Interferon-γ (NCBI GEO data, see S2 Table). Fold-changes from all three stimulations were averaged. MALAT1, miR146aHG and miR155HG data-points are highlighted. (PDF) [file pone.0193066.s001.pdf]

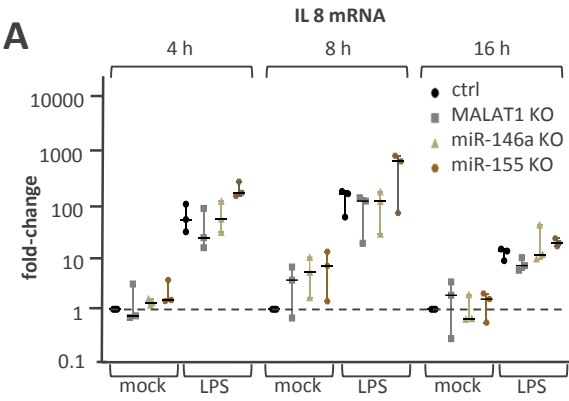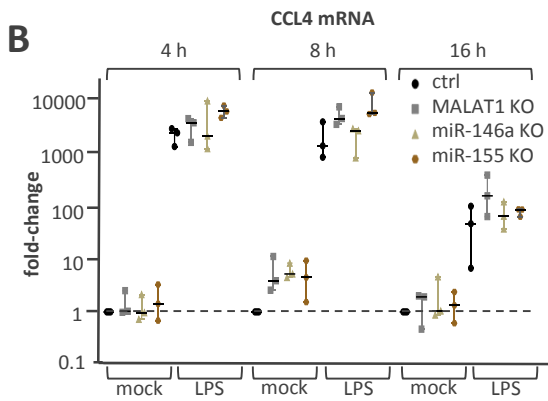

Supplement: S3 Fig — A) Analysis of IL8 mRNA expression changes in wild-type (“ctrl”) and the indicated ncRNA knockout cell clones comparing mock treatment and LPS stimulation (1 μg / ml) for the indicated durations. B) Same as A) but measuring CCL4 instead of IL8 mRNA expression changes. (PDF) [file pone.0193066.s003.pdf]

**A** miR146aHG co-expression network

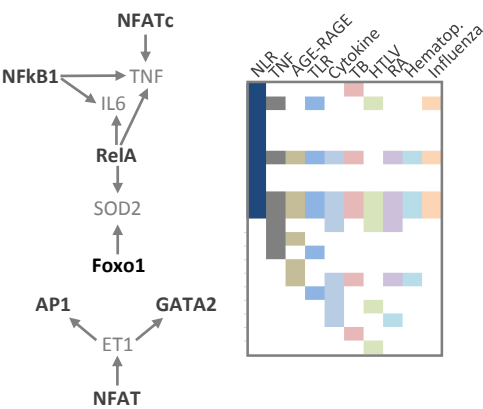

**B** miR155HG co-expression network

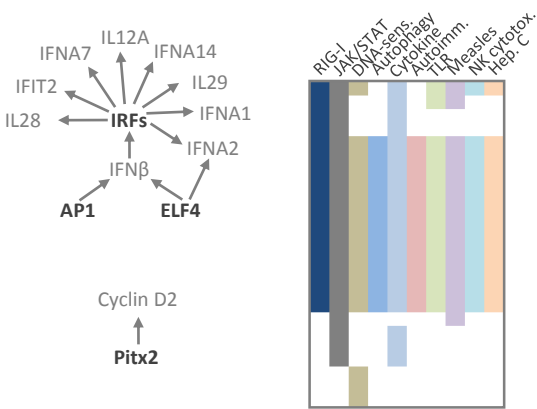

**C** MALAT1 co-expression network

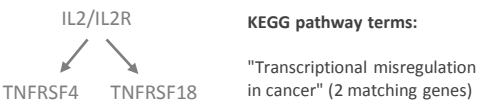

Supplement: S4 Fig — A) Transcription factor network (left panel; transcription factors: bold; transcription factor target genes: grey) and KEGG pathway analysis (right panel) of mRNAs co-expressed with the miR-146a host transcript in 45 mononuclear phagocyte RNA-Seq experiments (see S2 Table). Each square in the pathway heat-map represents a gene included in the respective pathway. Pathways are shown in different colours. Only pathways covering ≥ 5 genes from the input list were visualized. B) Same as A) but with miR-155 host transcript co-expressed mRNAs. C) Same as A) but with MALAT1 co-expressed mRNAs. Only one pathway (covering 2 genes from the input list) was identified. (PDF) [file pone.0193066.s004.pdf]

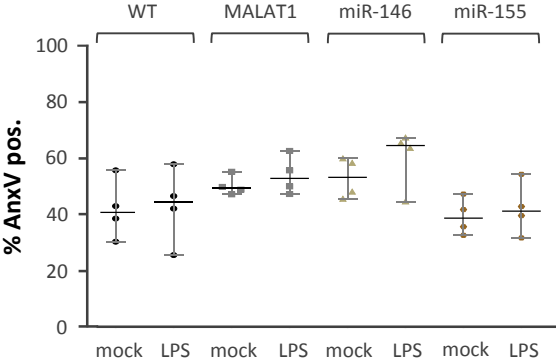

Supplement: S6 Fig — The percentage of AnnexinV (AnxV) positive cells either control-treated or stimulated with LPS (1 μg / ml) for 16 hours is shown. Genetic backgrounds are indicated above the panel (WT = wild-type). (PDF) [file pone.0193066.s006.pdf]
