# Supplementary material for: Cas9-mediated excision of proximal DNaseI/H3K4me3 signatures confers robust silencing of microRNA and long non-coding RNA genes
Source: PLoS One. 2018 Feb 16;13(2):e0193066. doi: 10.1371/journal.pone.0193066 (PMC5815609; doi:10.1371/journal.pone.0193066)

A

	miR-155		miR-146a		MALAT1	
Guide #	1	2	1	2	1	2
Off-targets	49	49	50	49	48	49
Genic	8	3	3	4	3	9

	random					
Guide #	1	2	3	4	5	6
Off-targets	50	50	37	49	50	50
Genic	5	3	7	6	5	6

B

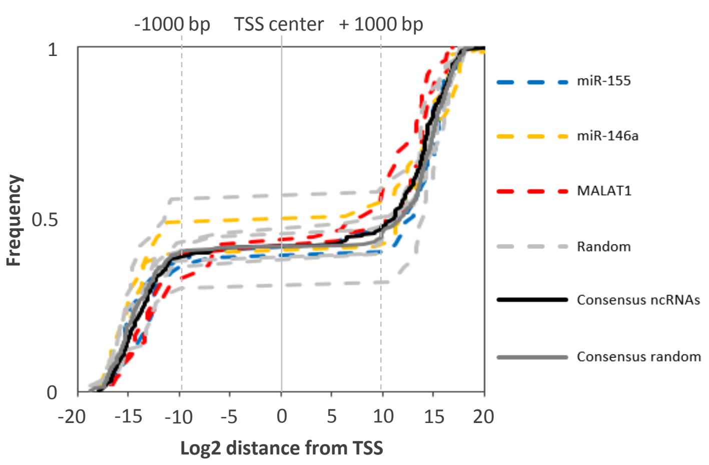

C

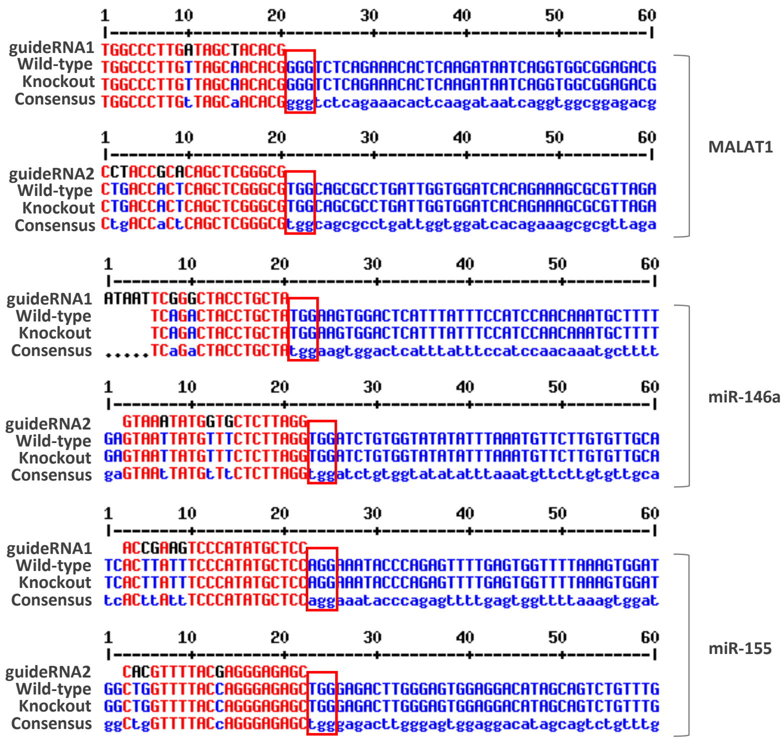

Supplement: S2 Fig — A) Upper panel: total number of off-targets (“off-targets”), and number of genic off-targets (“genic”) predicted by crispr.mit.edu for the guideRNA pairs (guide # 1 and 2) used to knockout miR146aHG, miR155HG or MALAT1. Lower panel: same as upper panel but for randomly selected guideRNAs number 1–6. B) Cumulative density plot representing the distance of predicted off-targets from A) to the next annotated transcript 5’ end (“TSS center)”. The guideRNA pairs for knockout of miR146aHG, miR155HG or MALAT1 are color-coded as indicated to the right (dashed lines). Random control guideRNA analysis are presented as grey dashed lines. The result of the combined analysis with data from all six guideRNAs targeting the miR146aHG, miR155HG or MALAT1 loci is shown as a solid black line (“Consensus ncRNAs”) and the combined analysis for all six random guideRNAs (“Consensus random”) as a solid grey line. C) Sanger sequencing results for the top predicted off-target loci of each guideRNA of the MALAT1, miR146aHG and miR155HG knockout constructs. Alignments aggregate the guideRNA sequence and the sequencing results obtained using wild-type or the respective knockout cells. The PAM motif is highlighted. Mutations are expected to occur ~3–4 nucleotides away from the PAM. (PDF) [file pone.0193066.s002.pdf]
