# Supplementary material for: Cas9-mediated excision of proximal DNaseI/H3K4me3 signatures confers robust silencing of microRNA and long non-coding RNA genes
Source: PLoS One. 2018 Feb 16;13(2):e0193066. doi: 10.1371/journal.pone.0193066 (PMC5815609; doi:10.1371/journal.pone.0193066)
Supplement: S5 Fig — Outputs of the “induced network” analysis for miR-146 host transcript (A), miR-155 host transcript (B) and MALAT1 host transcript (C) co-expressed mRNAs are shown. ConsensusPathDB legend is shown below the three co-expression network panels. (PDF) [file pone.0193066.s005.pdf]

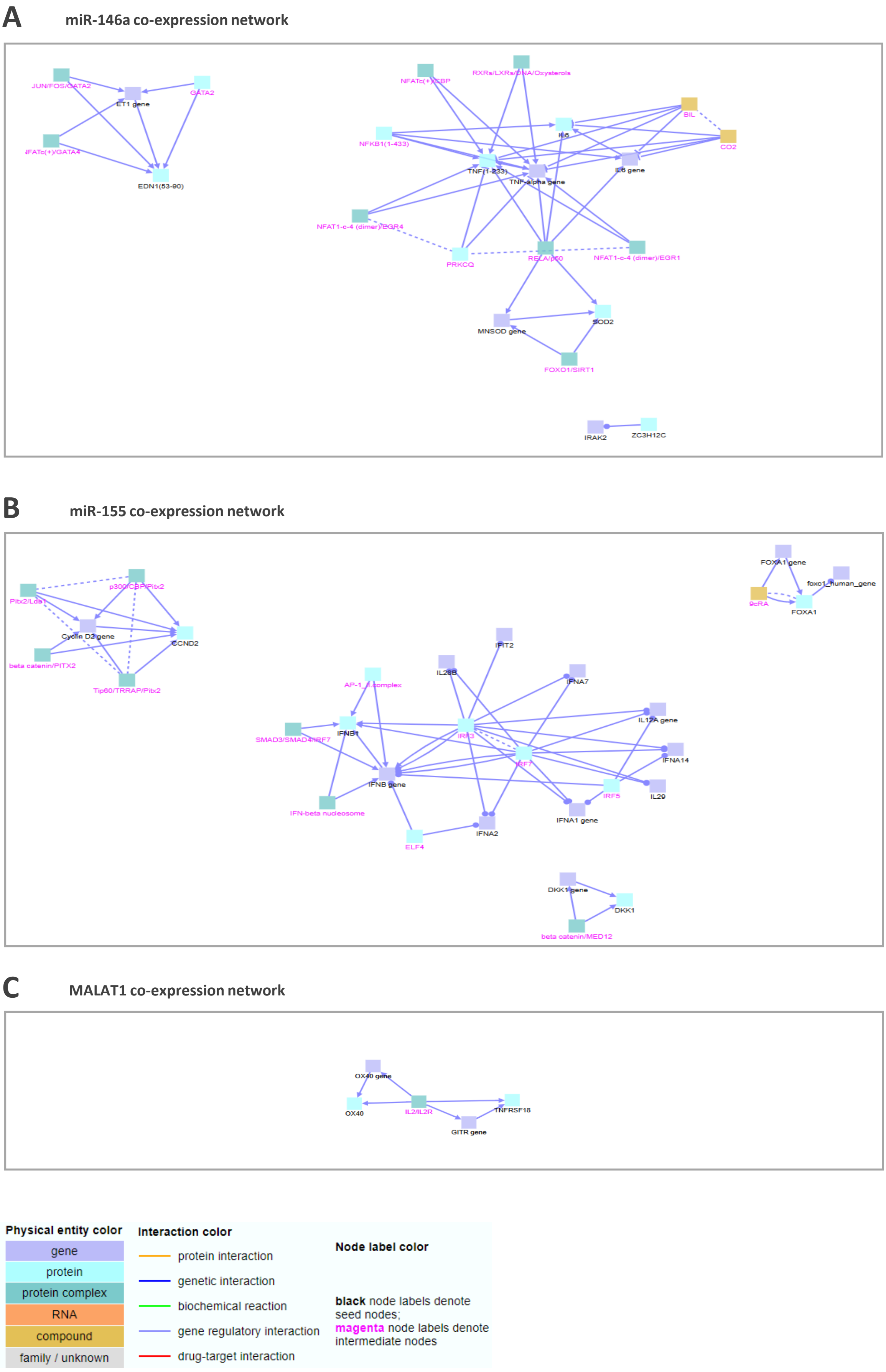
A
miR-146a co-expression network
JUN/FOS/GATA2
ET1 gene
GATA2
NFATc(+)/GATA4
EDN1(53-90)
NFATc(+)/CBP
RXRs/LXRs/DNA/Oxysterols
BIL
NFKB1(1-433)
IL6
CO2
TNF(1-233)
TNF-alpha gene
IL6 gene
NFAT1-c-4 (dimer)/EGR4
PRKCQ
RELA/p50
NFAT1-c-4 (dimer)/EGR1
MNSOD gene
SOD2
FOXO1/SIRT1
IRAK2
ZC3H12C
B
miR-155 co-expression network
Pitx2/Lda1
p300/CBP/Pitx2
Cyclin D2 gene
CCND2
beta catenin/PITX2
Tip60/TRRAP/Pitx2
FOXA1 gene
foxc1_human_gene
9cRA
FOXA1
IFIT2
IL28B
IFNA7
AP-1_jl_complex
IL12A gene
SMAD3/SMAD4/IRF7
IFNB1
IRF3
IRF7
IFNA14
IFNB gene
IRF5
IL29
IFN-beta nucleosome
IFNA1 gene
IFNA2
ELF4
DKK1 gene
DKK1
beta catenin/MED12
C
MALAT1 co-expression network
OX40 gene
OX40
IL2/IL2R
TNFRSF18
GITR gene
Physical entity color
gene
protein
protein complex
RNA
compound
family / unknown
Interaction color
protein interaction
genetic interaction
biochemical reaction
gene regulatory interaction
drug-target interaction
Node label color
black node labels denote seed nodes;
magenta node labels denote intermediate nodes
