# Supplementary material for: Cas9-mediated excision of proximal DNaseI/H3K4me3 signatures confers robust silencing of microRNA and long non-coding RNA genes
Source: PLoS One. 2018 Feb 16;13(2):e0193066. doi: 10.1371/journal.pone.0193066 (PMC5815609; doi:10.1371/journal.pone.0193066)
Supplement: S7 Fig — The sequence of the dual guideRNA pX458 cloning cassette used in the present study is provided. The functional elements of the cassette, to be cloned into the pX458 CRISPR vector via BbsI sites, are color-coded according to the explanations given below the sequence. GuideRNA sequences to be inserted are shown as “NNN…” and highlighted in yellow. (PDF) [file pone.0193066.s007.pdf]

pX458 dual guideRNA cassette:

▼
GATCGAGAAGACCTCACCGNNNNNNNNNNNNNNNNNNNNgttttagagctaGAAAtagcaagtta
aaataaggctagtccgttatcaacttgaaaaagtggcaccgagtcggtgcTTTTTTACTGATAGA
CTGGATCTGTTAGAATGAGCCTAGAGGGCCTATTTCCCATGATTCCTTCATATTTGCATATACGA
TACAAGGCTGTTAGAGAGATAATTGGAATTAATTTGACTGTAAACACAAAGATATTAGTACAAAA
TACGTGACGTAGAAAGTAATAATTTCTTGGGTAGTTTGCAGTTTTAAAATTATGTTTTAAAATGG
ACTATCATATGCTTACCGTAACTTGAAAGTATTTCGATTTCTTGGCTTTATATATCTTGTGGAAA
GGACGAAACACCGNNNNNNNNNNNNNNNNNNNNGTTTGGGTCTTCGATAGG
▲

BbsI binding motif ▲▼ BbsI cut sites GuideRNA sequence GuideRNA scaffold

Spacer sequence Second U6 promoter

GuideRNA sequences used in the present study:

MIR155HG	gRNA 1:	GGAGCATATGGGACTTCGGT
MIR155HG	gRNA 2:	GCTCTCCCTCGTAAAACGTG
MIR146aHG	gRNA 1:	TAGCAGGTAGCCCGAATTAT
MIR146aHG	gRNA 2:	CCTAAGAGCACCATATTTAC
MALAT1	gRNA 1:	CGTGTAGCTATCAAGGGCCA
MALAT1	gRNA 2:	CGCCCGAGCTGTGCGGTAGG
