# Supplementary material for: Cas9-mediated excision of proximal DNaseI/H3K4me3 signatures confers robust silencing of microRNA and long non-coding RNA genes
Source: PLoS One. 2018 Feb 16;13(2):e0193066. doi: 10.1371/journal.pone.0193066 (PMC5815609; doi:10.1371/journal.pone.0193066)
Supplement: S1 Table — DNA oligos used for qPCR, genomic PCR, Northern blot detection and Sanger sequencing are listed. (PDF) [file pone.0193066.s008.pdf]

Oligo ID	Sequence	Purpose
	**qPCR**	
OBS-712	GCTTCGGCAGCACATATACTAAAAT	hU6 forward
OBS-713	ATATGGAACGCTTCACGAATTTG	hU6 reverse
OBS-814	AGGTGCTACACAGAAGTGGATTCAG	hMalat 1 forward
OBS-815	CTTCCCGTACTTCTGTCTTCCAGT	hMalat 1 reverse
OBS-720	ATGGAGCAACAAGTGGTGTTCTC	hIL1β forward
OBS-721	TCAACACGCAGGACAGGTACAG	hIL1β reverse
OBS-017	ACTGAGAGTGATTGAGAGTGGAC	hIL8 forward
OBS-018	AACCCTCTGCACCCAGTTTTC	hIL8 reverse
OBS-733	AGCAAGCAAGTCTGTGCTGATC	hCCL4 forward
OBS-744	TGTGTCTCATGGAGAAGCATCC	hCCL4 reverse
	**Genomic DNA PCR**	
OBS-1288	GGTCAGCCTGAGACCACTTCT	hMalat 1 forward
OBS-1289	AACGCTAAGCAATATCTTAGTAACC	hMalat 1 reverse
OBS-1292	AGTGACTACATCTGCCTGGAAGC	hsa-miR-146a forward
OBS-1293	AAGTAATAGCCATAGTCTTCCAACC	hsa-miR-146a reverse
OBS-1290	TAGGCTTGTAGGATAAACTTGCC	hsa-miR-155 forward
OBS-1291	TAGATCTGCTGTGTGATGCTGG	hsa-miR-155 reverse
OBS-1922	ACATCAACTCTCCGGATGCC	Malat 1 off1 forward
OBS-1923	AGAGAGGACACGGCAGTCTG	Malat 1 off1 reverse
OBS-1952	ATATACCATCTCTCCTCGTC	Malat 1 off2 forward
OBS-1953	ATTGAGCTTTAGTCACTAAC	Malat 1 off2 reverse
OBS-1938	AGACAATTAATGGTCAATGAGTGC	miR-155 off1 forward
OBS-1939	TATGGTTGTAGGCTAGGCATTCTAAG	miR-155 off1 reverse
OBS-1942	ATCAATTGACTGCACATATGTGG	miR-155 off2 forward
OBS-1943	TCTAATCAGAGCAACTGAAGGAGG	miR-155 off2 reverse
OBS-1930	AAGCTTCCACTCTGGCTAAGC	miR-146a off1 forward
OBS-1931	CTCACACCTAATTTAGATACTGCGAG	miR-146a off1 reverse
OBS-1934	GAATCTCTTAATACATACATGCAGC	miR-146a off2 forward
OBS-1935	AGTTCCGGTTTCTACTTCCTC	miR-146a off2 reverse
	**Northern blot**	
OBS-1787	CTACCTGCACTGTAAGCACTTTG	hsa-miR-17
OBS-1618	ACCCCTATCACGATTAGCATTAA	hsa-miR-155
OBS-1619	AACCCATGGAATTCAGTTCTCA	hsa-miR-146a
	**Sanger sequencing**	
OBS-842	CTGGCCTTTTGCTCACATGT	pX458_forward
OBS-843	GTCTGCAGAATTGGCGCAC	pX458_reverse
