# Supplementary material for: Cas9-mediated excision of proximal DNaseI/H3K4me3 signatures confers robust silencing of microRNA and long non-coding RNA genes
Source: PLoS One. 2018 Feb 16;13(2):e0193066. doi: 10.1371/journal.pone.0193066 (PMC5815609; doi:10.1371/journal.pone.0193066)
Supplement: S2 Table — All RNA-Seq datasets deposited in the NCBI GEO pipeline and used in the present study are listed. (PDF) [file pone.0193066.s009.pdf]

Cell type	Monocytes	Monocytes	Macrophages	Macrophages	Dendritic cells	Dendritic cells
Dataset description	Mock, LPS stimulation or gram-pos. infection	Mock, LPS or IFNγ stimulation	Mock or gram-pos. infection	Mock, IFNγ or PAM3CSK4 stimulation	Mock or gram-pos. infection	Mock, CpG or IMQ stimulatio n
Datasets & SRR accession numbers	SRR1539216	SRR1282180	SRR1947529	SRR1910733	SRR1725922	SRR3229076
	SRR1539218	SRR1282181	SRR1947545	SRR1910734	SRR1725926	SRR3229077
	SRR1539219	SRR1282182	SRR1947552	SRR1910737	SRR1725930	SRR3229078
	SRR1539221	SRR1282195	SRR1947567	SRR1910738	SRR1725934	SRR3229082
	SRR1539222	SRR1282196	SRR1947590		SRR1725938	SRR3229083
	SRR1539224	SRR1282197	SRR1947603		SRR17259242	SRR3229084
	SRR1539244		SRR1947605			SRR3229088
	SRR1539246		SRR1947529			SRR3229089
	SRR1539247		SRR1947620			SRR3229090
	SRR1539249		SRR1947632			
